# Anesthetic management of external iliac artery transection in a morbidly obese patient with Klippel-Trenaunay-Weber syndrome: a case report

**DOI:** 10.1186/s40981-023-00609-9

**Published:** 2023-04-13

**Authors:** Naoki Hirai, Hirotaka Kinoshita, Masato Kitayama, Tetsuya Kushikata, Kazuyoshi Hirota

**Affiliations:** 1 Department of Anesthesiology, Hirosaki University Graduate School of Medicine, 5 Zaifu-Cho, Hirosaki, 036-8562 Japan; 2 Department of Perioperative Medicine for Community Healthcare, Hirosaki University Graduate School of Medicine, 5 Zaifu-Cho, Hirosaki, 036-8562 Japan; 3 Department of Perioperative Stress Management, Hirosaki University Graduate School of Medicine, 5 Zaifu-Cho, Hirosaki, 036-8562 Japan

**Keywords:** Klippel-Trenaunay-Weber syndrome, Obesity, Regional saturation of oxygen, Regional anesthesia, Airway management, Hemodynamic system

## Abstract

**Background:**

We report the anesthetic management of an external iliac artery transection in a morbidly obese patient with Klippel-Trenaunay-Weber syndrome (KTWS).

**Case presentation:**

A 47-year-old man with KTWS was scheduled for a right external iliac artery transection. Preoperative CT showed a right external iliac artery aneurysm, a right superficial femoral artery aneurysm, and developed collateral vessels. General anesthesia was maintained with desflurane, remifentanil, and rocuronium bromide. After the transection of the right external iliac artery, the regional saturation of oxygen (rSO_2_) value of the right femoral did not decrease. There was no significant hemodynamic change before or after the transection. A non-ultrasound-guided rectus abdominis sheath block was performed due to the many collateral vessels. After extubation, the patient did not complain of postoperative pain.

**Conclusions:**

In the transection of lower-extremity blood arteries under laparotomy in patients with KTWS, rSO_2_ monitoring, hemodynamic monitoring, and combined regional anesthesia could be useful.

## Background

Klippel-Trenaunay-Weber syndrome (KTWS) is characterized by the triad of a port-wine stain, varicose veins, and bony and soft-tissue hypertrophy of an extremity [1]. Venous malformations are known to progress rather than regress, and these malformations can cause ischemia and thrombotic painful events. The estimated incidence of KTWS is 2–5 cases in 100,000 and is found equally in both sexes [2].

There are few reports about the anesthetic management of patients with KTWS [3, 4], which requires attention to the possibility of excessive circulatory dynamics in order to avoid bleeding and deep vein thrombus formation, plus the careful consideration of regional anesthesia due to the development of collateral vessels [3]. Herein, we report the successful anesthetic management of an external iliac artery transection in a morbidly obese patient with KTWS; regional saturation of oxygen (rSO_2_) oximetry was administered for the patient’s bilateral femoral region, and a hemodynamic monitoring system and regional anesthesia were used.

## Case presentation

We have obtained a written informed consent from the patient and his family for the publication of this case report.

**Fig. 1:**
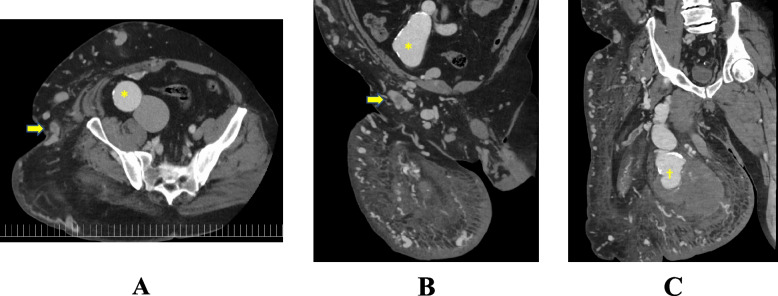
Preoperative CT image. **A** Transverse plane: an external iliac artery aneurysm (*), numerous collateral vessels, dilatation of vessels, and venous thrombosis (arrow) were observed. **B** Coronal plane: an external iliac artery aneurysm (*), numerous collateral vessels, dilatation of vessels, and venous thrombosis (arrow) were observed. **C** Coronal plane: a superficial femoral artery aneurysm (†) and numerous collateral vessels were observed

A 47-year-old man (height 174 cm, weight 148 kg, body mass index 48.9 kg/m^2^) with KTWS was admitted to the hospital due to right femoral swelling. When he was 22 years old, he had undergone the amputation of his right femur due to a congestive ulcer. Thereafter, he had been treated for high-output heart failure. His legs gradually became edematous and developed movement restrictions. Therefore, he was scheduled for right external iliac artery transection. Preoperative computed tomography (CT) demonstrated a right external iliac artery aneurysm, a right superficial femoral artery aneurysm, developed collateral vessels, and intravenous thrombosis (Fig. 1). Preoperative platelet count, fibrinogen, and D-dimer were 221 × 10^3^/µL, 239 mg/dL, and 43.6 µg/mL, respectively.

Besides standard monitors, we used direct arterial blood pressure and central venous pressure in preparation for the potential massive bleeding. A FloTrac™ sensor (Edwards Lifesciences, Irvine, CA, USA) was used to monitor hemodynamic changes. A central venous pressure catheter was inserted into the right internal jugular vein in preparation for the potential massive bleeding. General anesthesia was induced by an intravenous injection of propofol 240 mg, remifentanil 0.5 µg/kg/min, and rocuronium bromide 50 mg. Despite predicted difficult airway due to morbid obesity and obstructive sleep apnea, mask ventilation and tracheal intubation with video laryngoscopy (McGRATH™, Covidien Japan, Tokyo) were uneventfully performed following adequate preoxygenation. The general anesthesia was maintained with desflurane 5%, remifentanil 0.1–0.25 µg/kg/min, and rocuronium bromide.

An INVOS™ 5100C regional oximetry system (Covidien Japan) was placed on the patient’s bilateral femoral region to detect ischemia on the affected side before and after the transection of the right external iliac artery. After transection of the right external iliac artery, the rSO_2_ of the right femur did not decrease (Table 1). We were concerned about preload reduction via the arteriovenous fistula after the transection, but there was no significant change in the patient’s cardiac output or stroke volume variation.

**Table 1:** Changes in regional oxygen saturation and hemodynamics before and after transection of the right external iliac artery

	**Before transection**	**After transection**
rSO2, left/right; %	95/72	88/77
CO, L/min	8.9	7.3
SVV, %	3.1	4.3
HR, bpm	61	59
sBP/dBP, mmHg	116/65	107/56
CVP, mmHg	14	13

*CO* Cardiac output, *CVP* Central venous pressure, *dBP* Diastolic blood pressure, *HR* Heart rate, *rSO2* Regional saturation of oxygen, *sBP* Systolic blood pressure, *SVV* stroke volume variation

**Fig. 2:**
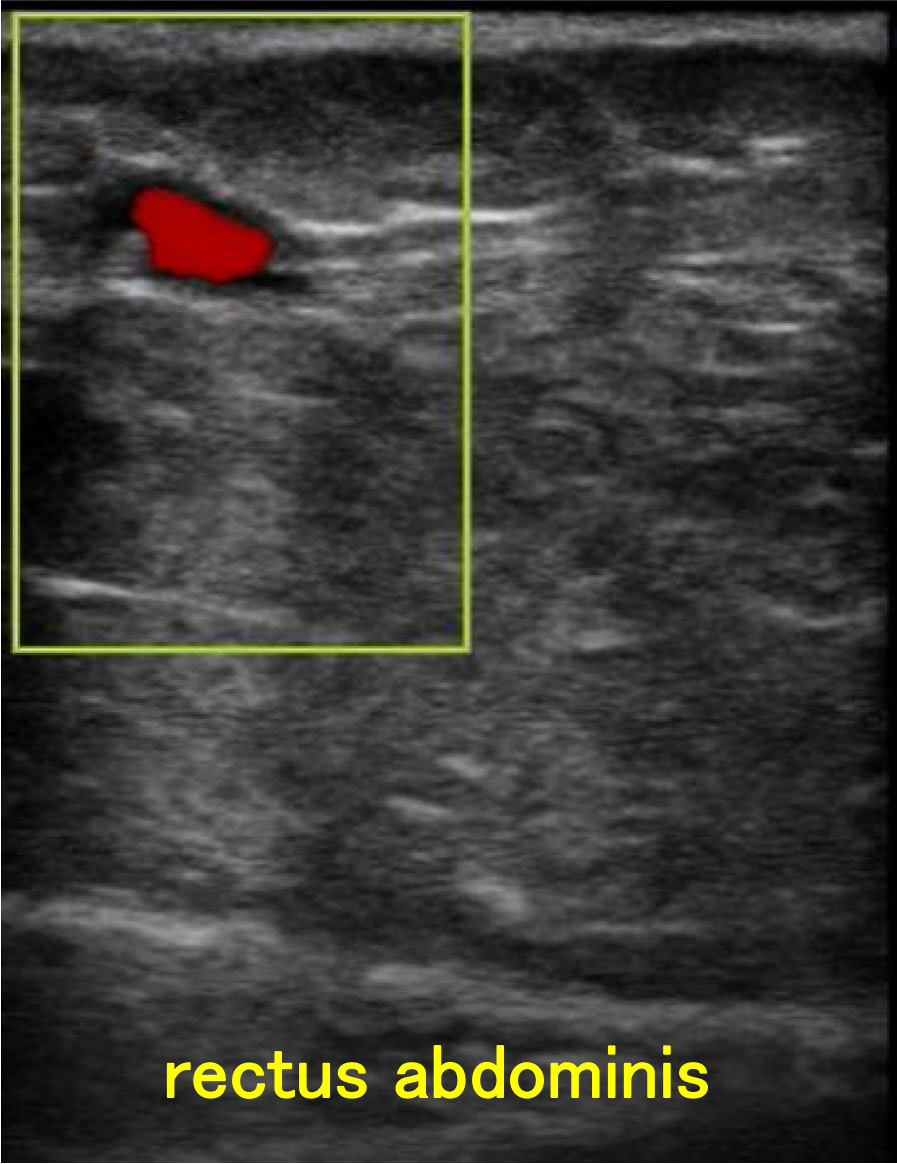
Intraoperative superficial echography image. Subcutaneous collateral vessels were observed

The surgery was performed through a lower midline abdominal incision. To reduce the need for opioids for postoperative analgesia, we planned to perform a rectus abdominis sheath block (RSB) and a posterior transversus abdominis plane block (TAPB) just after the general anesthetic induction. However, superficial echography showed many collateral vessels in the abdominal surface (Fig. 2). We asked surgeon to perform a non-ultrasound-guided RSB at the closing of the patient’s abdomen. In addition to an RSB, postoperative analgesia was performed via an intravenous injection of morphine 15 mg and flurbiprofen 50 mg. The anesthesia time was 326 min, and the blood loss was 120 g. The patient was extubated after being admitted to the postanesthesia care unit (PACU). He did not complain of postoperative pain during the PACU stay.

## Discussion

KTWS is a rare congenital malformation with severity ranging from very minimal to life-threatening. Vascular malformations in patients with KTWS can rapidly progress when complicated by increased circulating blood volume, blood stasis, or infection. Individuals with KTWS must therefore try to avoid weight gain [5]. The vascular malformation restricted the present patient’s physical activity, which further exacerbated his obesity during treatment.

We used the INVOS™ 5100C system placed on the patient’s bilateral femoral region to detect ischemia on the affected side before and after the transection of the right external iliac artery. There are several reports that the measurement of rSO_2_ on the legs is useful to detect ischemia in patients with acute compartment syndrome and surgical resection of common iliac artery aneurysms [6, 7]. Thus, rSO_2_ monitoring could be useful to confirm ischemia and perfusion via collateral blood vessels in the affected limb.

The causes of intraoperative massive bleeding in patients with KTWS have been associated with not only vascular malformations but also coagulation disorders such as disseminated intravascular coagulation and Kasabach-Merritt coagulopathy [3, 8]. In addition, the disruption of the vessel wall due to circulatory fluctuations further contributes to bleeding. Securing central venous access and the preparation of transfusion products are essential in the anesthesia of KTWS patients. In addition to the above, we used a FloTrac™ sensor to manage fluid volume and circulatory fluctuations due to the presence of the femur arteriovenous fistula. As a result, there was no significant change in the patient’s cardiac output or stroke volume variation after the transection of the right external iliac artery.

We selected desflurane and remifentanil to prevent postoperative oversedation and respiratory depression associated with morbid obesity [9, 10]. Combined regional anesthesia for obstructive sleep apnea of obesity is useful [11], but it is difficult to perform regional anesthesia for KTWS patients due to the development of collateral vessels [3]. There are a few reports regarding neuraxial anesthesia for KTWS patients [3, 8, 12], but there is a single report about the use of an abdominal wall block for a KTWS patient [4]. After our patient’s preoperative CT images confirmed the development of subcutaneous collateral vessels, we did not perform an ultrasound-guided peripheral nerve block. An RSB was performed after the preoperative ultrasound to confirm the absence of collateral vessels within the rectus abdominis muscle. In fact, the postoperative analgesia was sufficient with an RSB and an intravenous injection of morphine 15 mg and flurbiprofen 50 mg. Thus, image diagnostics including CT and superficial echography should always be used in patients with KTWS when regional anesthesia is used.

KTWS patients can have potentially difficult intubation due to the frequent presence of soft tissue hypertrophy, upper airway angiomas, and facial anomalies [13]. In addition to these features, our patient had sleep apnea associated with obesity. Although we anticipated a difficult airway for the patient, mask ventilation and tracheal intubation with video laryngoscopy were easy without airway bleeding. In their 2011 review of 136 patients with KTWS, Barbara and Wilson reported that difficulty with intubation was not encountered [3].

In conclusion, we achieved successful management of an external iliac artery transection in a morbidly obese patient with KTWS. In the transection of lower-extremity blood arteries under laparotomy in patients with KTWS, rSO_2_ monitoring, hemodynamic monitoring, and combined regional anesthesia could be useful.

## Data Availability

Please contact the corresponding author for data requests.

## Abbreviations

CT: Computed tomography
ICU: Intensive care unit
KTWS: Klippel-Trenaunay-Weber syndrome
rSO_2_: Regional saturation of oxygen
PACU: Postanesthesia care unit
RSB: Rectus abdominis sheath block
TAPB: Transversus abdominis plane block
